# Designer drugs 2015: assessment and management

**DOI:** 10.1186/s13722-015-0024-7

**Published:** 2015-03-25

**Authors:** Michael F Weaver, John A Hopper, Erik W Gunderson

**Affiliations:** The University of Texas Health Science Center at Houston, 1941 East Road, BBSB 1222, 77054 Houston, TX USA; St. Joseph Mercy Hospital, 5333 McAuley Drive, Suite R-3009, 48197-1014 Ypsilanti, MI USA; The University of Virginia, Center for Wellness and Change, 1007 East High Street, 22902 Charlottesville, VA USA

**Keywords:** Designer drugs, Legal high, Bath salts, Cathinones, Mephedrone, Methylone, Methylenedioxypyrovalerone, Synthetic cannabinoids, Spice, 25I-NBOMe, N-bomb, Hallucinogens

## Abstract

Recent designer drugs, also known as “legal highs,” include substituted cathinones (e.g., mephedrone, methylone, and methylenedioxypyrovalerone, often referred to as “bath salts”); synthetic cannabinoids (SCs; e.g., Spice); and synthetic hallucinogens (25I-NBOMe, or N-bomb). Compound availability has evolved rapidly to evade legal regulation and detection by routine drug testing. Young adults are the primary users, but trends are changing rapidly; use has become popular among members of the military. Acute toxicity is common and often manifests with a constellation of psychiatric and medical effects, which may be severe (e.g., anxiety, agitation, psychosis, and tachycardia), and multiple deaths have been reported with each of these types of designer drugs. Clinicians should keep designer drugs in mind when evaluating substance use in young adults or in anyone presenting with acute neuropsychiatric complaints. Treatment of acute intoxication involves supportive care targeting manifesting signs and symptoms. Long-term treatment of designer drug use disorder can be challenging and is complicated by a lack of evidence to guide treatment.

## Introduction

There is growing international concern about the synthetic analogs of controlled substances being manufactured and distributed to circumvent drug laws and evade interdiction. These compounds are referred to as “designer drugs” or “legal highs” [1] (see Table 1). They are substances with psychotropic effects that are intentionally marketed and distributed for recreational use by exploiting inadequacies of existing controlled substance legislation [2]. Nonchemists can easily synthesize the compounds with readily available raw materials, or they can obtain the synthetic compounds directly [3]. The chemicals are often packaged with labels that inaccurately describe product contents, which may vary substantially regarding chemical content and concentration [4]. Labels often include the phrase, “not for human consumption,” in an attempt to avoid legal risk. Designer drug use has expanded in the past decade—especially among young adults—leading to significant problems for some users.

**Table 1:** **Designer drugs**

**Drug class**	**Chemical name**	**Chemical origin**	**Slang names**
Stimulant	Mephedrone	Cathinone	Bath salts (Ivory Wave, Vanilla Sky), meow-meow, M-Cat
	Methylone		
	Methylenedioxypyrovalerone (MDPV)		Sextacy
	Naphyrone		NRG-1
Cannabinoid	JWH-018; JWH-073; JWH-250	Laboratory of J.W. Huffman	Spice, K2, K9, Aroma, herbal highs, Scooby Snax
	CP 47,497; CP 47,497-C8; CP 59,540; cannabicyclohexanol	Pfizer laboratory	
	HU-210	Hebrew University laboratory	
	UR-144	CB2 receptor agonist	
	Oleamide	Fatty acid	
	XLR-11, AKB-48		
	AM-2201, AM-694		
Hallucinogen	25I-NBOMe	Free University of Berlin	N-bomb, Solaris, Smiles, Cimbi-5
	25B-NBOMe		
	25C-NBOMe		

Although the emerging designer drug trend was initially recognized by increasing calls to US poison control centers [5], the incidence of designer drug problems in emergency departments (EDs), hospitals, and other medical settings is largely unknown. Only a small percentage of those using designer drugs will come into contact with the health-care system, but consequences of use can be severe. Familiarity with designer drugs can help clinicians recognize common adverse reactions and life-threatening consequences. This article will focus on three newer designer drugs: substituted cathinones (commonly referred to as “bath salts”), synthetic cannabinoids (SCs; e.g., “Spice”), and synthetic hallucinogens (e.g., “N-bomb”).

### Epidemiology of emerging designer drugs

Designer drug use is most prevalent among young adults, primarily males in their mid to late 20s, but ranging from teens to adults 40 years of age [6-9]. Those who use designer drugs tend to be single and have lower levels of education and income [10,11]. The use of SC products may be higher in select subpopulations, such as in regular cannabis users [12], university students [13], and dance club attendees [7]. Among high school seniors, the annual prevalence of SC consumption was 11 percent in 2011 and 2012 [11]. Annual prevalence rates among high school seniors dropped to 8 percent in 2013, but remained more prevalent than any illicit drug except cannabis (annual use of cannabis remained unchanged) [11]. Bath salt use appears comparably lower than SC, with 1 percent use among the same sample [11]. Overall use of hallucinogens remains very low in the United States, and the epidemiology of synthetic hallucinogens is not currently captured in national surveys.

The growing popularity of designer drugs relates to factors such as novelty, marketing, and accessibility. Designer drug packaging is colorful and attractive, with enticing names for the products to attract younger individuals to try them [14,15]. Designer drugs are sold without age restriction, which also makes them attractive to younger individuals. Widespread availability, including purchase via the Internet, has contributed to expanded use. Marketing designer drug products as “legal high” alternatives may contribute to the perception of greater safety or purity compared to traditional illicit drugs, which could promote increased consumption [16]. Risk factors for adolescent experimentation with and problems resulting from designer drug use include parents with substance use disorders (SUDs), poor family relationships, poor discipline, or high family conflict; adolescents involved with foster care or the criminal justice system are also at risk [17].

In response to rising designer drug use and consequences, a series of state and federal initiatives have been enacted during the past several years prohibiting the manufacture, sale, and possession of many designer compounds. Although designer drug use has persisted despite regulatory efforts [11,12,18], there may be a national trend toward reduced consumption of some designer drugs [11]. Use appears to be growing in some subpopulations—including the US military—perhaps to evade detection by urine drug screening [18]. Designer drug use is especially prevalent among those in the military who abuse other substances. Patients presenting for consequences of designer drug use will frequently be using more than a single drug.

### Bath salts

#### Pharmacology

Most designer stimulants are derivatives of cathinone, the primary active alkaloid in the natural herbal stimulant khat (*Catha edulis*). Three of the primary substituted cathinones sold as bath salts include: mephedrone, methylenedioxypyrovalerone (MDPV), and methylone [2]. The designer substituted cathinones are part of the larger family of stimulants that includes amphetamine, methamphetamine, and methylenedioxymethamphetamine (MDMA, or “ecstasy”).

Substituted cathinones appear to increase extracellular levels of the monoamine neurotransmitters dopamine, norepinephrine, and serotonin via facilitation of extracellular release and reuptake inhibition [3,19,20]. These neurochemical effects likely account for similar sympathomimetic, subjective, and reinforcing effects of the substituted cathinones. The pharmacology and product effects (such as increased alertness, tachycardia, and potential for psychosis) appear similar to stimulants such as amphetamines and cocaine.

#### Acute clinical effects

Most designer stimulants are taken intranasally but may be consumed orally or via intravenous or intramuscular injection [7,9]. Mephedrone is not suitable for smoking. Anecdotally, the effects generally start about 10-20 minutes after dosing, peak at 45-90 minutes, last 2-3 hours, and then decrease over 6-12 hours. Users may consume multiple doses during a session to prolong the desired effects [17].

Commonly reported effects that overlap with cocaine, amphetamine, and MDMA include increased energy, alertness, concentration, sexual stimulation, empathy, talkativeness, mood enhancement, euphoria, and decreased appetite [6,17].

#### Adverse psychiatric effects

Most users report intermittent adverse effects [7,13]. Acute toxicity may be associated with larger binge consumption and exposure to multiple substances. Acute agitation is a hallmark of toxicity [21]. Psychosis may be pronounced, with patients experiencing paranoia, hallucinations (primarily visual), and delusions.

Repeated dosing of substituted cathinone likely leads to tolerance [22], which is indicated indirectly by the association between frequency of use and greater amount consumed [7]. Binges have been reported with significant successive dosing of mephedrone [7]. Withdrawal effects reported among chronic users include: tiredness, insomnia, difficulty concentrating, irritability, depression, and nasal congestion [6]. Some users experience a dependence syndrome, with cravings and compulsive use [6,7,13,23]. The liability for development of a severe diagnosable SUD from use of chronic substituted cathinone could be quite high [21].

#### Adverse physiologic effects

Bath salts were largely responsible for a doubling in annual stimulant/sympathomimetic-related toxicology cases reported, from 6 percent in 2010 to 12 percent in 2011 [5]. Commonly reported effects include diaphoresis, palpitations, muscle tension or spasms, and bruxism (jaw clenching) [6,17]. Most individuals exhibit autonomic hyperactivity on exam (e.g., tachycardia, hypertension). Nasal-specific adverse effects include epistaxis and sore nasal passages, mouth, and throat [13].

Sympathomimetic toxicity is manifested by neurological and cardiovascular clinical features. The use of bath salts has been associated with cardiac arrhythmias and myocarditis [18]. Significant hyponatremia has been reported with mephedrone use (similar to that seen with MDMA), which is likely due to a combination of sweating, electrolyte loss, and antidiuretic hormone secretion [24]. More serious renal impairment includes acidosis and acute renal failure associated with rhabdomyolysis. Deaths have been reported with mephedrone and MDPV [21].

In addition to the cathinone effects, contaminants could play a role in adverse effects. Product analysis studies have demonstrated adulteration with benzocaine, lidocaine, procaine, caffeine, or even controlled drugs such as cocaine, amphetamine, ketamine, and piperazine compounds [4,8,25]. Adulterants with stimulant properties could potentiate the effects of bath salts and raise toxicity risk by increasing the sympathetic effects or chances of cardiac arrhythmias [26].

Empirical or prospective data are limited regarding long-term adverse physiological effects of substituted cathinone use. However, neurotoxicity is plausible (e.g., monoamine depletion, neuronal degradation) along with development of physiological dependence among regular users, which is manifested by tolerance and a withdrawal syndrome [21].

### Synthetic cannabinoids

#### Pharmacology

The primary cannabinoid in cannabis is delta-9-tetrahydrocannabinol (THC), a partial agonist at the CB1 receptor, which is located throughout the human body, especially the central nervous system. SCs used recreationally may be full or partial CB1 agonists that were originally synthesized for research purposes in different university laboratories. Frequently, the SC-containing products used recreationally include individual or mixtures of different SC compounds sprayed on psychoactively inert pulverized plant matter of virtually unknown content to resemble potpourri or incense [27]. The term Spice is now generally applied to all products containing SC, regardless of branding [2]. Compared to THC, SCs are often more potent and efficacious CB1 agonists and may have a longer half-life, all of which may lead to greater cannabinomimetic toxicity [28]. There is substantial variability in product composition and wide concentration ranges for SC [29], which can also add to the risk of toxicity.

#### Acute clinical effects

SCs are primarily smoked via a joint, bowl, or water pipe, although they can be consumed orally or intranasally [28]. Acute effects are similar to cannabis, including alteration in mood, conjunctival injection, and tachycardia [30]. Effects are reported to start within 10 minutes after inhalation, and most effects appear to dissipate 2-6 hours after use [30,31].

#### Adverse psychiatric effects

Adverse psychological effects are common with the use of SC products [12] and may include anxiety, trouble thinking clearly, agitation, paranoia, and delusions [32]. Reports indicate that SC can provoke acute psychosis—which appears more likely in users with underlying biologic vulnerability due to family history of psychosis—as well as worsen pre-existing chronic psychotic disorders [18,33,34]. Psychotic symptoms can persist for a significant time, from 1 week to 5 months in reported cases [33].

Some regular users of cannabis may use SC as a substitute to relieve cannabis withdrawal symptoms, likely indicating cross-tolerance between SC and THC [12,30]. SCs appear to serve as a sufficient cannabis substitute, especially when cannabis is unavailable [12]. Case reports have documented withdrawal symptoms after SC product use, as well as a dependence syndrome, similar to those seen with cannabis [35].

### Adverse physiologic effects

Commonly reported side effects include dry mouth, lightheadedness, and headache [12,36]. Other unwanted negative physiological effects include diaphoresis, tremors, dystonia, and dyspnea [32]. Tachycardia is common with SC use (similar in cannabis users), due potentially to reduced peripheral vascular resistance and the subsequent need to maintain cardiac output by increasing heart rate, rather than due to a direct sympathetic effect. The tachycardia may be severe, along with hypertension and chest pain [18]. One case report of significant bradycardia with chest pain has also been reported [37].

Several SC compounds (specifically JWH-018, JWH-073, and AM-2201) have been implicated as a cause of cannabinoid hyperemesis syndrome, which is a chronic disorder that was originally characterized among chronic cannabis users who experienced cyclic episodes of vomiting and abdominal pain relieved by bathing or showering with hot water [38]. However, cannabis-related hyperemesis syndrome is quite rare. To the extent that SC might be more likely to cause nausea and vomiting, such symptoms could help to differentiate intoxication between the two [39].

Severe SC-related toxicity requiring emergency treatment has included seizures, acute renal failure, and myocardial infarction [30,40-43]. Deaths have been reported with SC due to a cardiac ischemic event and extreme anxiety resulting in suicide [44].

There are no studies of the long-term effects of SC. Smoking SC products results in the inhalation of burned unidentified plant material as well as SC, which may have adverse effects on the pulmonary system, so some sources recommend vaporization instead of smoking as a cannabinoid delivery method [45]. Additionally, JWH-018 may be a carcinogen [46]; anecdotal data indicate the development of tolerance and a withdrawal syndrome with chronic use [30].

### Synthetic hallucinogens

#### Pharmacology

Synthetic designer hallucinogens gained popularity after the 1991 publication of Alexander Shulgin’s book, *PIHKAL, A Chemical Love Story*. PIHKAL, an acronym for “Phenethylamines I Have Known and Loved,” details the synthesis of over 200 psychedelic compounds [47]. The “2C” series of hallucinogenic phenethylamines, first described by Shulgin, share a similar chemical structure; the term “2C” is derived from the two carbon molecules between the benzene ring and the amino group. These compounds have a similar structure to MDMA and produce hallucinations through serotonergic stimulation.

The substituted phenethylamine 4-iodo-2,5dimethoxy-N-(2-methoxybenzyl) phenethylamine (25I-NBOMe) is a relatively new derivative of the 2C series of phenethylamines. 25I-NBOMe is one of several N-benzyl phenethylamines that have become popular since October 2011 when the U.S. Drug Enforcement Administration issued a temporary Schedule I status on many of the compounds marketed as bath salts [48]. 25I-NBOMe is a highly potent, high-affinity agonist of the serotonin 2a (5HT2a) receptor [49] that was originally synthesized for research on the serotonin receptor [50]. Other hallucinogenic NBOMe drugs include 25C-NBOMe and 25B-NBOMe [51].

#### Acute clinical effects

Use of 25I-NBOMe in both liquid and powder form has been reported, with many potential routes of administration, including inhalation of vapor, nasal insufflation, oral ingestion, sublingual/buccal administration, and intravenous injection [51-53]. The most common use is oral/sublingual/buccal, but nasal insufflation is not unusual [51]. When administered by the oral/oral mucosal route, 25I-NBOMe is ingested as a pill or absorbed as powder or on blotter paper. Use of the drug generally occurs in a single administration of a small quantity, about 0.1 gram, or “cap.” Clinical effects can occur rapidly after nasal use and generally peak in 20 minutes. A wide duration-of-action range of 3-13 hours has been reported [51]. In reported cases of clinical toxicity, agitation persisted for several days [50,52].

The reported effects of 25I-NBOMe are similar to those of prototypical serotonergic hallucinogens such as lysergic acid diethylamide or psilocybin [54]. Users report hallucinations with a varying degree of stimulating effects. Depersonalization has been reported as well. In contrast to prototypical serotonergic hallucinogens, 442 users responding to an Internet survey reported that 25I-NBOMe had greater “negative effects while high,” but with more “value for money” [51].

#### Adverse psychiatric effects

In addition to the anticipated visual and auditory hallucinations, many users experience psychiatric consequences, prompting them to access medical services. Some of these consequences include delirium, agitation, aggression, violence, paranoia, dysphoria, severe confusion, and self-harm [50,52,53,55,56]. Some patients have presented with a serotonergic or sympathetic toxidrome consisting of an “excited delirium” with severe agitation, aggression, and violence [47]. In one case, a reportedly hallucinogen-naïve 19-year-old man died from a multiple-story fall after ingesting 25I-NBOMe and developing paranoid and bizarre behavior [55]. In another fatal case, a 21-year-old male driver who ingested 25I-NBOMe developed sudden rage, pulled his car off the road, and began to destroy the inside of the vehicle before dying from an unknown cause [56].

#### Adverse physiologic effects

Tachycardia, hypertension, and mydriasis are frequently described in the few clinical reports of 25I-NBOMe users [50,52,53]. Hyperreflexia and clonus have also been reported in several cases [52,53]. Seizures occurred in many of the cases that eventually required medical attention [52,53]. Severe toxicity has included hyperthermia, pulmonary edema, and death from trauma [50,52,55,56]. In one report of a fatal exposure, a 15-year-old girl became unresponsive after ingesting 25I-NBOMe outside a rave; on arrival at a local hospital she was in asystole with a rectal temperature of 39.9°C [56]. Long-term physiologic effects are not known.

### Drug testing challenges

Urine or serum toxicology screens are unable to detect all of the designer drugs that have been synthesized, posing a major diagnostic and monitoring challenge for clinicians. Although laboratory testing is expanding, widespread standardized designer drug testing is not yet available in most clinical practice settings and laboratories. The analytical challenge is compounded by heterogeneity in designer drug product contents, concentration, and chemical constituents, all of which may vary between and within products [57].

Illicit manufacturers have demonstrated remarkable flexibility in altering the psychoactive components of designer drugs to evade regulation and detection [2,58]. It is common practice by drug designers to modify functional groups, change substitutions, and alter moieties of substances in a rapid and iterative process to evade legal restriction [2]. This practice also poses significant challenges for detection of compounds or metabolites through urine drug screening.

Individuals frequently report that the lack of detection on standard urine drug screening tests is a reason for designer drug product use [21]. For example, populations under criminal justice supervision may use designer drugs to evade detection by probation officers. Among the US military, where most soldiers referred for addiction treatment are identified through urine drug screening, SCs are consumed by those seeking cannabis-like mood-altering effects, but with much lower risk of detection [59].

Even though most emerging designer drugs will not be picked up on routine urine drug screening in a health-care setting, collection of urine is still valuable clinically to test for unreported, co-occurring substance use. A general laboratory screening battery of urine or serum should be sent to screen for common drugs of abuse [26]. This helps the clinician to be aware of potential toxicity due to drug interactions, or to the need for closer or prolonged monitoring due to the presence of other, nondesigner substances. When comprehensive designer drug testing is unavailable or pending, familiarity with the most common designer drugs and other substances of abuse in a given locality can help clinicians rapidly recognize intoxication and begin management of serious complications.

### Screening and assessment

The challenges with designer drug analytic testing necessitate vigilance to recognize use. Young adults are the most common demographic among those seeking emergency medical services related to designer drug use [60]. Hence, clinicians should consider direct inquiry about designer drug use, particularly among young adults presenting for acute medical care with signs or symptoms that could indicate substance-related toxicity. Since designer drugs are not detected by routine drug screens, health-care providers relying solely on laboratory testing may be misled to believe that illicit drugs have not been used [32]. Conversely, the presence of routinely detectable illicit substances does not rule out the presence of designer drugs, since polysubstance use is typical in the population using designer drugs. Clinicians should be alert for inconsistencies between observed and expected intoxication syndrome from a self-reported or detected class of drugs. Such discrepancies could indicate recent designer drug use.

Clinicians can be alert for clinical clues based on variations in patient presentation that may help identify designer drug use (see Table 2). Conjunctival injection is an indicator of SC intoxication, as well as other cannabis products. Some patients presenting for emergency treatment may still have the package that contained the designer drug. This can be examined for possible identification of common brand names for a specific class of designer drug (see Table 1) and, potentially, any remaining content can be sent to a laboratory for analysis. Internet sites may be helpful for identification of specific substances ingested due to their rapidly changing appearance [61]. However, the lack of research-based information on the adverse effects of designer drugs has led to the emergence of a range of websites that may or may not provide accurate information [62]. The presence of paraphernalia such as a pipe for smoking could indicate designer drug or other smokable drug use, and a strong smell of perfume or cologne may be an attempt to mask the smell of recent smoking.

**Table 2:** **Indicators of designer drug use**

**Body system**	**Finding**	**Medical indication**	**Drug (s)**
General	Hyperthermia	Intoxication	Synthetic hallucinogens, bath salts
Head & neck	Conjunctival injection	Recent use	Synthetic cannabinoids
	Smoky chemical smell on breath	Recent smoking	Any smoked designer drug
	Epistaxis	Intranasal use	Bath salts, synthetic hallucinogens
	Nasal septal perforation	Intranasal use	Bath salts
	Poor dentition	Inadequate oral hygiene	Bath salts
	Jaw clenching, teeth grinding (bruxism)	Intoxication	Bath salts
Cardiac	Tachycardia	Recent use	Any designer drug
	Hypertension	Recent use	Any designer drug
	Chest pain	Cardiac ischemia, myocarditis	Bath salts, synthetic cannabinoids
Renal	Acute kidney injury	Recent use	Synthetic cannabinoids
Gastrointestinal	Nausea, vomiting	Recent use or withdrawal syndrome	Synthetic cannabinoids
	Enlarged and/or tender liver	Acute hepatitis	Any injected designer drug
Musculoskeletal	Muscle spasms	Intoxication	Bath salts
	Limb swelling and pain	Rhabdomyolysis	Bath salts, synthetic hallucinogens
Skin	Diaphoresis	Recent use	Bath salts
	Ecchymosis	Recent use or intoxication	Synthetic hallucinogens
	Fresh needle marks, track marks	Injection drug use	Any injected designer drug
Neurologic	Clonus	Recent use	Synthetic hallucinogens
	Seizures	Intoxication	Bath salts, synthetic hallucinogens, synthetic cannabinoids
Psychiatric	Agitation	Recent use	Any designer drug
	Hallucinations	Recent use	Any designer drug
	Psychosis	Intoxication	Any designer drug

Routine inquiry about designer drug use is likely prudent, particularly among patients with a history of SUD, those who are undergoing mandated urine testing (e.g., criminal justice supervisees), or among those who have reported a history of designer drug use of a different chemical class [63]. Different classes of designer drugs may be used concurrently, which could increase the incidence of adverse effects and toxicity. It is helpful for clinicians to ask about specific products by name, or perhaps “synthetics” in general, since patients may not be aware of designations used by medical personnel, or of different street names for similar products. For each affirmative response, follow-up questions should be asked about frequency, patterns of use, and subjective effects. Careful inquiry about subjective effects could help provide insight into the designer drug class, particularly when the brand-compound association is less well established and with wide variation in contents. Although Table 1 lists brand names along with the designer drug compound or class, the list is not comprehensive; there are likely thousands of different trade-name brands sold internationally [29].

Further clinical inquiry should include specific questions about factors associated with designer drug use and the potential consequences, whether related to medical, interpersonal difficulties, or financial or legal problems. Chronic designer drug use may lead to physiologic dependence with tolerance and abstinence-related withdrawal, as well as a designer drug use disorder [21,29]. Comprehensive inquiry about such factors regarding the patient’s designer drug use helps the clinician make an initial determination about potential severity and provides insight into treatment needs.

Among patients presenting for acute medical complications of designer drug use, routine laboratory testing should include—in addition to standardized urine drug testing—a complete blood cell count and complete metabolic panel. Cardiac enzymes should be obtained if cardiac symptoms are present. Creatine phosphokinase is helpful if rhabdomyolysis is suspected on the basis of severe muscle spasms, swelling and pain in the extremities, or severe seizures. Additional diagnostic studies may be selected on the basis of the initial presentation.

### Management of acute intoxication

No specific antidotes are available for designer drug toxicity. Activated charcoal is not useful unless there has been significant oral ingestion. Most nonpsychiatric symptoms appear self-limited and resolve within one to several days with supportive treatment. Unpleasant psychological effects of acute intoxication, such as anxiety, agitation, or paranoia may be managed with supportive treatment. Placing the distraught user in a quiet environment and maintaining gentle contact is often sufficient until the acute effects subside [64].

Psychosis due to synthetic cannabinoids (SC) and 25I-NBOMe intoxication have been managed with monitored observation [52,65]. For psychopathological clinical features, benzodiazepines have been used to treat anxiety, agitation, and seizures [8,52,53,65]. Antipsychotics are second-line agents for agitation [8], due to the lowered seizure threshold with use of cathinone and phenethylamine designer drugs [66]. Sedation may be required if the patient is markedly agitated and at risk for harm to self or health-care staff [63]. Since some designer drug-associated psychosis may be severe and require prolonged inpatient treatment, psychiatric consultation is indicated, in particular for those with persistent symptoms.

Abrupt discontinuation of stimulants or hallucinogens does not cause gross physiologic sequelae, so they are not tapered off or replaced with a cross-tolerant drug during medically supervised withdrawal [26]. Abrupt discontinuation of SC could result in withdrawal symptoms such as nausea and irritability, similar to that with cannabis cessation. However, there is no indication for pharmacologic replacement (e.g., dronabinol), since SC withdrawal is not life-threatening. Patients can be treated with supportive care by intravenous fluids and antiemetics if necessary. If marked psychiatric symptoms persist longer than one or more weeks after discontinuation, the patient should be evaluated carefully to determine whether he or she has a co-occurring primary psychiatric disorder, which then should be treated with specific therapy [67]. Treatment of prolonged anxiety, depression, or psychosis is the same as when these conditions are not associated with recent designer drug use.

For a significant number of patients, the high level of illness severity warrants admission to critical care. Intoxicated patients should be placed initially on continuous cardiac monitoring with pulse oximetry and frequent neurological assessments [63]. Adequate administration of intravenous fluids is encouraged to assure good urine output, as these patients often are dehydrated [63]. Fluid administration in the presence of rhabdomyolysis can help prevent acute renal failure. Intensive monitoring allows for early detection and intervention for serious consequences, such as myocardial infarction.

Patients may present with concurrent ingestion of drugs with different pharmacological profiles, including both stimulant and depressant drugs. Clinicians should be alert for an unexpected response to a therapeutic intervention or to a change in patient presentation as one type of designer drug wears off and ongoing intoxication with another class of designer drug is revealed. This may require some flexibility in treatment due to changes in mental or cardiovascular status.

## Treatment of designer drug addiction

Hospitalization for the adverse effects of designer drugs affords an excellent opportunity (teachable moment) for advising patients to decrease their substance use and for engaging them in treatment [68]. Health-care provider awareness and patient education are cornerstones of public health initiatives [2] to confront new challenges presented by designer drugs. Simple admonitions to stop are sometimes helpful if the diagnosis is made early, but in most cases are insufficient [69]. Many patients who use designer drugs may be ambivalent about changing behavior, so the clinician should express empathy without confrontation, which shows respect for the patient’s autonomy. Providing appropriate, accurate information about the potential risks of designer drugs and encouraging healthy choices can help patients make the best informed decision about changing behavior. Physicians should involve the patient proactively in the process of problem-solving, while reminding the patient of responsibility for all actions. The responsibility of the practitioner is to motivate the patient to seek recovery from designer drug use instead of blaming the patient for being unmotivated to change [69]. Accurate information about the relative risks and unknown harms of these products helps a patient make an informed choice about continuing to use particular products, to make a quit attempt, or to seek more specific addiction treatment.

Although prospective treatment data are limited, once a designer drug use disorder diagnosis is made, acute and long-term treatment is likely necessary [70]. Recovery from SUD in general is possible, and those who are treated have less disability than those who remain untreated [71]. Long-term treatment of designer drug use disorders likely involves similar components to that of other types of addiction treatment, including behavioral components, such as individual and group counseling with cognitive-behavioral therapy, motivational enhancement therapy, and 12-Step self-help group facilitation. Family members should be considered as part of the treatment program, in particular when treating adolescents or young adults. Unfortunately, pharmacologic treatment data to guide management of those with designer drug use disorders are unavailable.

Patients identified with SUD in the ED or hospital inpatient setting should be provided with information linking them to local community addiction treatment resources. In the United States, physicians certified in the treatment of addictive disorders can be found through the American Society of Addiction Medicine (www.asam.org) or the American Academy of Addiction Psychiatry (www.aaap.org). At times, it may be more expedient and cost effective to refer the patient to a nonphysician counselor [64], which can be found through the National Association for Alcohol and Drug Abuse Counselors’ website (www.naadac.org). Substance abuse treatment services in the United States can also be located via the Substance Abuse and Mental Health Services Administration Behavioral Health Services Treatment Locator (http://findtreatment.samhsa.gov).

Treatment of designer drug SUD is challenging for several reasons. Designer drugs consist of several different classes of substances, which vary in their psychological and physiological effects. Treatment is often difficult due to the young age of most users and the possibility of concurrent polysubstance use. The pattern of use is usually intermittent in social settings, so it may be perceived as less of a problem. Clinicians should be knowledgeable of and prepared to provide treatment for very different combinations, such as what occurs with club drug use [72]. A treatment environment with a supportive structure can be helpful. Addiction treatment is cost effective [73], and even multiple episodes of treatment are worthwhile. It can be very rewarding for any health-care practitioner to assist a patient who was impaired by addiction return to normal functioning in society.

## Conclusions

Bath salts, SC, and 25I-NBOMe are relatively new designer drugs that continue to remain popular drugs of abuse, especially among young adults. Though chemically different, they are similar in that they are groups of synthetic analogs that continue to change in attempts to avoid legal issues and detection on drug tests. They are also similar in that adverse reactions are common, especially clinically significant psychotic reactions. Detection of these drugs with urine tests is challenging, so clinicians should consider designer drug use in young adults with agitation and psychosis. Treatment is primarily supportive, and benzodiazepines may be beneficial. When those who use designer drugs come into contact with the health-care system, it is important for clinicians to provide linkage to additional, specific treatment for the substance use disorder.
